# Multidimensional effects of the Xin’an Jianpi Tongbi Formula on self-perception of patients with rheumatoid arthritis: focusing on the mediating role of systemic inflammation index

**DOI:** 10.3389/fmed.2026.1756862

**Published:** 2026-01-30

**Authors:** Yang Li, Jian Liu, Yue Sun, Yuan Wang, Yuedi Hu, Jianting Wen, Yiming Chen, Chengzhi Cong

**Affiliations:** 1 Department of Rheumatology, The First Affiliated Hospital of Anhui University of Chinese Medicine, Hefei, Anhui, China; 2 Anhui Provincial Key Laboratory of Applied Basic and Clinical Translational Research in Traditional Chinese Medicine Rheumatology, Hefei, Anhui, China; 3 First Clinical Medical School, Anhui University of Chinese Medicine, Hefei, Anhui, China

**Keywords:** cohort study, rheumatoid arthritis, self-perception of patients, systemic inflammation, Xin’an Jianpi Tongbi Formula

## Abstract

**Objective:**

To investigate the effects of the Xin’an Jianpi Tongbi Formula (XAJPF), composed mainly of Huangqin Qingre Chubi Capsule (HQC) and Xinfeng Capsule (XFC), on the systemic inflammation and self-perception of patients (SPP) composite outcomes in rheumatoid arthritis (RA), and innovatively analyze the mediating effect of systemic inflammation index.

**Methods:**

This retrospective cohort study was conducted in the Department of Rheumatology of the First Affiliated Hospital of Anhui University of Chinese Medicine from August 2023 to February 2025. The study used admission and discharge as primary time points, grouping 935 RA patients based on their baseline median neutrophil-to-lymphocyte ratio (NLR) levels and employing propensity score matching to control for intergroup confounders. Exposure was defined as receiving XAJPF treatment (divided into high/low exposure groups based on the type). Multimodal analyses, including logistic regression, association rules, mediation analysis, and sensitivity analysis, were used to comprehensively evaluate the effects of XAJPF on the primary outcomes (SPP scores) and secondary outcomes [NLR and systemic inflammation response index (SIRI)].

**Results:**

After matching, baseline characteristics were balanced between groups. Higher inflammatory status was associated with worse SPP scores. Compared with the non-exposure group, XAJPF exposure demonstrated superior efficacy in improving SPP outcomes and systemic inflammation levels, without causing hepatic or renal injury. Multivariable-adjusted logistic regression identified XAJPF as an independent protective factor against worsening PF, GH, VT, MH, VAS, PGA, PhGA, CPRI-RA, SDS, SDH, and SDSSD, with odds ratios of 0.24, 0.60, 0.54, 0.50, 0.44, 0.46, 0.54, 0.56, 0.43, 0.29, and 0.49 (all *P*<0.05), with exposure-response relationships observed. Mediation analysis indicated that NLR and SIRI mediated 5.1–14.4% of XAJPF’s improvement effects on VAS, PhGA, and SDSSD scores. Subgroup and sensitivity analyses underscored the robustness of these effects.

**Conclusion:**

This study establishes the pivotal role of NLR and SIRI in the inflammation-perception axis of RA and confirms them as partial mediators of the multidimensional therapeutic effects of XAJPF. This provides a novel perspective for understanding the clinical application of the Chinese compound.

## 1 Introduction

Rheumatoid arthritis (RA) is a chronic, systemic autoimmune inflammatory disease characterized by persistent synovial inflammation and progressive bone destruction (1). Its symptoms include joint swelling, pain and limited mobility. The global prevalence of RA has been reported to be approximately 0.3–1% and is more prevalent in women than men (2). Recent surveys have revealed that in China, a country with a large population, the prevalence of RA has risen rapidly in recent years, leading to an escalation of economic burdens and social issues (3). Furthermore, significant challenges exist in ensuring both accessibility and standardization of diagnosis and treatment. Existing therapeutic options manage RA from two perspectives: one is non-steroidal anti-inflammatory drugs (NSAIDs) and glucocorticoids (GCs) aimed at symptom management, and the other is disease-modifying anti-rheumatic drugs (DMARDs), which promote remission by primarily suppressing autoimmune activity and slowing or preventing joint degeneration (4). Although these treatments have shown positive effects on patient prognosis, they are associated with serious adverse events such as infections and gastrointestinal reactions (5, 6). Therefore, the development of novel drugs that combine potent anti-inflammatory activity and good tolerability for the treatment of RA is imperative.

In recent years, natural herbs, particularly traditional Chinese medicine (TCM) formulas, have been widely used in the treatment of RA owing to their advantages of complex composition, multi-targeting pathways, and fewer adverse effects (7). Based on the theoretical principle of “tonifying qi and strengthening the spleen, clearing heat and dispelling dampness” in TCM, it is advocated to apply Xin’an Jianpi Tongbi Formula (XAJPF), which mainly consists of Huangqin Qingre Chubi Capsule (HQC) and Xinfeng Capsule (XFC), in the treatment of patients with RA. This herbal formula possesses comprehensive effects such as tonifying qi, strengthening the spleen, clearing heat, dispelling dampness, resolving blood stasis, and clearing collaterals. It is worth mentioning that both HQC and XFC have been granted national invention patents and approved by the Food and Drug Administration of Anhui Province (HQC: Patent No.: ZL201110095718.X, Registration Number: Z20200001; XFC: Patent No.: ZL201310011369.8, Registration Number: Z20050062). In particular, XFC has been included in the Guidelines of Diagnosis and Treatment of Rheumatoid Arthritis Disease and Syndrome Combination as an industry-wide recommendation for the use of key formulas with evidence-based medical evidence (recommendation level: B) (8). Previously, our group has conducted systematic studies on the preparation process, fingerprinting, and pharmacokinetics of HQC and XFC, which fully verified the scientific production technology, stable chemical composition, and strict quality standards (9–11). Previous large-sample cohort studies and randomized controlled trials have emphasized the independent efficacy of HQC and XFC in improving joint pain and reducing the risk of hospital readmission in RA patients, respectively, with no adverse effects (12, 13). Moreover, pharmacological studies using animal models have demonstrated potent anti-inflammatory effects of HQC and XFC (14, 15). Nevertheless, how these findings translate into the multidimensional patient-reported benefits observed in clinical practice remains to be elucidated.

The fragmentation of traditional inflammatory markers from the subjective experience of patients has long constrained the scientific interpretation of the efficacy of TCM in RA. Based on the characteristics of inflammatory arthritis including RA, the International Consortium for Health Outcome Measurement (ICHOM) proposed a standardized outcome set for patients including pain, activity limitation, fatigue, and assessment of overall mood and physical health impact, and recommended its implementation globally (16). Self-perception of patients (SPP) is based on this standardized outcome assessment framework, integrating patient-reported outcomes (PROs) across multiple dimensions of overall function, somatic pain, disease activity, and health-related quality of life, and innovatively incorporating a TCM evidence-specific assessment system (17). A previous study by our group confirmed that RA patients commonly present SPP abnormalities in the form of anxiety/depression and reduced quality of life, the extent of which is significantly correlated with the level of inflammation and the state of disease activity (18). It is noteworthy that there remains a lack of composite outcome assessment systems that systematically integrate systemic inflammation with patients’ multidimensional self-perception. Therefore, the construction of systemic inflammation-SPP composite endpoints can more sensitively capture the multi-targeted efficacy characteristics of XAJPF, which is an important outcome indicator to measure the disease status of RA patients.

In recent years, composite indices derived from routine blood tests have garnered significant attention due to their accessibility, low cost, and high reproducibility. Our team’s prior research confirmed that the platelet-to-lymphocyte ratio is significantly correlated with SPP outcomes in RA patients (19). Nonetheless, the pathological core of RA is more directly reflected in acute inflammation driven by neutrophil infiltration, immune imbalance caused by lymphocyte dysfunction, and chronic inflammation and bone destruction mediated by monocyte/macrophage activation (20, 21). Consequently, the neutrophil-to-lymphocyte ratio (NLR) directly reflects the core balance between pro-inflammation and immune regulation, while the systemic inflammation response index (SIRI) further incorporates information on monocytes, which are closely associated with bone erosion (22, 23). Nevertheless, the specific roles of NLR and SIRI within the RA SPP framework, particularly their potential mediating role in the process by which XAJPF improves SPP, have not been systematically elucidated.

Since the heterogeneity of RA patients’ response to treatment may be partly due to differences in baseline inflammation levels, it is difficult to adapt traditional single outcome models to the multi-target modulation properties of TCM. Therefore, building upon the preliminary work undertaken, we designed a case-control cohort study, i.e., NLR pre-stratification to construct a dual reference system of “exposure subgroup comparison within the case group and external low NLR control,” to reveal the intrinsic association between the composite outcomes of systemic inflammation and SPP, and to assess the net efficacy of XAJPF in the target subpopulation (high-inflammation load), with an emphasis on eliminating the interference of the low inflammation state on the interpretation of the efficacy. This study aims to reveal the dual association pattern of XAJPF in modulating systemic inflammation and improving patient’s feeling, and to provide evidence-based decision-making basis for TCM treatment of RA.

## 2 Materials and methods

### 2.1 Data sources and study population

**FIGURE 1:**
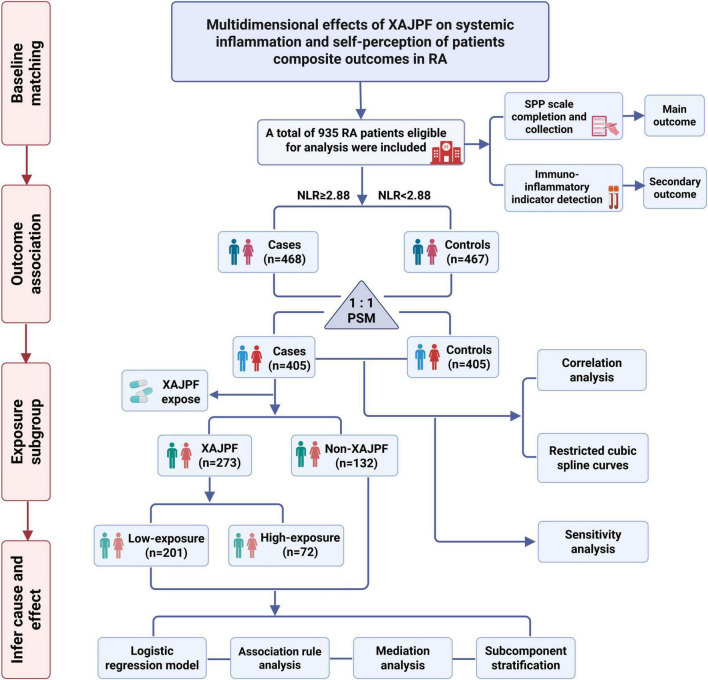
Flow chart for cohort study and analysis.

This retrospective cohort study enrolled RA patients admitted to the Rheumatology Department of the First Affiliated Hospital of Anhui University of Chinese Medicine between August 2023 and February 2025. RA diagnosis was determined based on the International Classification of Diseases (ICD)-10 code (M06.900). Inclusion criteria for all patients were: (1) strict adherence to the 2010 American College of Rheumatology (ACR) and European League Against Rheumatism (EULAR) RA classification criteria (24); (2) age ≥ 18 years; (3) complete and traceable medical records. Exclusion criteria included: (1) concomitant severe cardiovascular, hematologic, respiratory, or endocrine disorders; (2) minors, pregnant, or lactating patients; (3) concomitant other rheumatic diseases such as systemic lupus erythematosus or ankylosing spondylitis; (4) missing NLR, SIRI index, or key covariate data. This real-world retrospective study ultimately enrolled 935 eligible RA cases. The primary observation period spanned from hospital admission to discharge. Detailed procedures for all research and analysis are illustrated in Figure 1.

The study protocol was registered on the International Traditional Medicine Clinical Trial Registry (ITMCTR) on September 20, 2024 (Registration No.: ITMCTR2024000692). The study strictly adhered to the ethical principles of the Declaration of Helsinki and received review approval from the Ethics Committee of the First Affiliated Hospital of Anhui University of Chinese Medicine (Approval No.: 2024AH-92).

### 2.2 Outcome event definitions

The SPP scale was used as our RA-PROs and primary outcomes, including the MOS item short form health survey (SF-36), Visual analog scale (VAS), Patients’ general assessment of disease activity based on a visual analog scale (PGA), Physician’s general assessment of disease activity based on a visual analog scale (PhGA), Chinese patient-reported activity index with rheumatoid arthritis (CPRI-RA), Self-assessed anxiety scale (SAS), Self-assessed depression scale (SDS), Syndrome score of Dampness-Heat (SDH), Syndrome score of Dampness Stagnancy Due to Spleen Deficiency (SDSSD), and Syndrome score of Blood Stasis (SBS). Among them, the SF-36 scale is widely recommended to be used as an important reference tool for evaluating physical and mental health in clinical studies of RA (25, 26), which measures eight dimensional components, including physical function (PF), role physical (RP), body pain (BP), general health (GH), vitality (VT), social function (SF), role emotional (RE), and mental health (MH). The SF-36 scale applies a score of 0–100 as a general health rating index, and equal to or greater than 50 is defined as the normal range. The VAS score is the most realistic and intuitive pain symptom rating result based on patients’ self-visualization, with the following scoring criteria: 0 for no pain, 1–3 for mild pain, 4–6 for moderate pain, and 7–10 for severe pain. CPRI-RA is a unique RA-PRO scale that evaluates the degree of disease activity from the perspective of Chinese patients with RA, and it provides a complementary, objective, and quantitative tool for evaluating the efficacy of the clinical trials of RA (27, 28). The SDS and SAS are important complementary tools for assessing the psychological status of RA patients and have been widely promoted worldwide (29), with their standardized scores having a cut-off value of 50 points. In particular, the TCM evidence scores, here referred to as SDH, SDSSD, and SBS, correspond to the three types of evidence manifestations in RA patients, i.e., Damp-Heat (manifested by burning and swelling of the joints, and red tongue with yellow fur), Dampness Stagnancy Due to Spleen Deficiency (e.g., lethargy and nausea, and loose stools), and Blood Stasis (tingling of the joints), respectively. They transform subjective TCM evidence into quantifiable data by calculating the total score of the entries, which is a characteristic complement and innovation to the ICHOM-recommended clinical endpoints and provides a standardized tool for TCM efficacy assessment (12, 30). After the patient’s informed consent, two professionally trained rheumatology medical practitioners conducted a face-to-face interview with the patient. During the completion process, the physicians were responsible for providing detailed, easy-to-understand instructions to the patients, while the patients were asked to circle the answer to each question that best described their current feelings. Once the scale assessment was completed, a two-person independent entry and validation mechanism was implemented to ensure data integrity.

Secondary endpoints of the study were NLR, SIRI, erythrocyte sedimentation rate (ESR), hypersensitive C-reactive protein (hs-CRP), rheumatoid factor (RF), and anti-cyclic citrullinated peptide antibody (CCP) before and after treatment. The core indices of interest in this study, NLR and SIRI, were calculated according to the following formulas: (1) NLR = neutrophil count (10^9^/L)/lymphocyte count (10^9^/L); (2) SIRI = [neutrophil count (10^9^/L) × monocyte count (10^9^/L)]/lymphocyte count (10^9^/L). Additionally, to assess the safety of XAJPF, particularly with regard to potential hepatic and renal toxicity, we routinely monitored alanine aminotransferase (ALT), aspartate aminotransferase (AST), creatinine (CREA), and blood urea nitrogen (BUN) levels during treatment. All data were extracted from the inpatient medical record system of the First Affiliated Hospital of Anhui University of Chinese Medicine. Extraction was performed by two rheumatology physicians trained in standardized procedures using standardized data extraction forms.

### 2.3 Identification of exposure factors

The definition of exposure was aligned with the previous studie to ensure comparability (19). XAJPF exposure was identified from the cohort entry to the discharge date of the TCM use record, which here mainly includes HQC and XFC use. The detailed composition of the two herbal capsules and the key bioactive components identified in previous studies are summarized in Supplementary Table 1 (9–11). Among XAJPF users, they were further categorized into two subgroups based on the type and amount of use: low exposure (HQC alone or XFC alone) and high exposure (combination therapy with HQC and XFC). The conventional dosage for both HQC and XFC was three capsules three times per day, each containing approximately 0.4 g of quantified components. The primary treatment and observation period in this study spanned the entire hospitalization from admission to discharge, with a median duration of approximately 2–3 weeks. The implementation and documentation of the TCM treatment regimen were managed by rheumatology clinicians to ensure traceability of treatment adherence.

### 2.4 Collection of covariates

Covariates included gender, age, body mass index (BMI), smoking, drinking, course of disease, and individual past comorbidities. BMI was calculated based on weight and height, applying the formula: BMI = weight (Kg)/height (m^2^). Smoking and drinking status were assessed by applying self-report. Comorbidities were defined as diseases identified at the time of hospitalization. The Charlson-Deyo comorbidity index (CCI), a method that takes into account both the number and severity of 17 predefined comorbidities, was calculated here to quantify the comorbidity burden of the participants.

### 2.5 Statistical analysis

All statistical analyses were conducted in accordance with guidelines for observational studies and methodological specifications. The analysis plan was pre-specified prior to data inspection to ensure objectivity. Data management and primary analyses were performed using IBM SPSS Statistics (version 26.0). Advanced modeling, including restrictive cubic spline regression and causal mediation analysis, was implemented using R software (version 4.3.2).

#### 2.5.1 Descriptive analysis

The Shapiro-Wilk test was applied to test the normality of all quantitative parameters. Continuous variables were expressed as medians (interquartile spacing), and comparisons were made using nonparametric tests if comparisons were made between two groups. Non-parametric tests of paired samples were employed to compare changes in outcome indicators before and after treatment. Categorical variables were expressed as counts and percentages, and trend comparisons were made by the Pearson Chi2 test. Differences were considered statistically significant when *P* < 0.05.

#### 2.5.2 Propensity score matching

Differing from prior research, this cohort was constructed with a focus on defining cases and controls based on baseline NLR levels, followed by propensity score matching (PSM). PSM mathematically transforms observational studies into randomized studies by using propensity score values to find one or more individuals with the same or similar background characteristics for each individual to serve as a control (31). The basic formula is as follows:


e(X)=P(Z=1|X)

The propensity score model incorporates the following covariates, selected on the basis of their potential confounding effects on the relationship between NLR stratification and outcomes: gender, age, BMI, smoking, drinking, course of disease, and CCI. Based on the estimated propensity scores for each group, nearest neighbor matching was performed at a 1:1 ratio. The caliper width was set to 0.20 times the standard deviation of the propensity scores to ensure balanced matching quality (32).

#### 2.5.3 Correlation analysis

Spearman correlation analysis is a non-parametric measure of degree that describes the relationship between two variables (x, y) using a monotonic function. The formula is calculated as follows:


$$r\,s=1-\frac{6\,\sum {\left(R\,i-Q\,i\right)}^{2}}{n\,\left(n^{2}-1\right)}$$

The Mantel test is a method proposed by Nathan Mantel to determine the correlation between 2 sets of distance measurement matrices (33). We used Spearman’s correlation analysis and Mantel’s test to examine the correlations between all inflammation indicators and SPP outcomes and baseline variables, and to generate correlation matrix heat maps and networks. These were achieved in R 4.3.2 by utilizing the “linkET” and “ggplot2” packages.

#### 2.5.4 Restricted cubic spline curves

To examine the dose-response relationship (linear or nonlinear) between systemic inflammatory outcomes and SPP outcomes, we performed Restricted Cubic Spline (RCS) regression analyses of NLR, SIRI, and SPP each score. The dependent variable, SPP each score, was converted from a continuous variable to a dichotomous variable based on threshold or median. The RCS curves were plotted through logistic regression models where the number of nodes was determined based on the Akaike Information Criterion (AIC) minimum. We implemented this model by utilizing the “rms” package in R 4.3.2.

#### 2.5.5 Logistic regression modeling

Binary logistic regression analysis was applied to assess the correlation between the use of XAJPF and improvement in SPP outcomes. First, the dependent variable, the degree of improvement in SPP scores, was divided according to the median of the difference between pre- and post-treatment and converted into a dichotomous category for analysis. The independent variable XAJPF exposure was assessed both as a dichotomous variable and further divided into levels of exposure as an ordinal variable. Model 1 (crude model) was unadjusted. Model 2 (partially adjusted model) was adjusted for sex, age, BMI, smoking, drinking, course of disease, and CCI. Model 3 (fully adjusted model) was adjusted for the same variables as Model 2 and for indicators of inflammation that were associated with SPP at baseline with potentially confounding effects. Correlation results were expressed as odds ratios (ORs) and 95% confidence intervals (CIs) in the three predefined models, and Model 3 results were visualized as forest plots.

#### 2.5.6 Association rule analysis

The association rule analysis based on Apriori algorithm was applied to mine the association combination law between the type of XAJPF exposure and the improvement of RA system inflammation-SPP outcome. The third-order measures of support, confidence and enhancement were calculated to assess the strength of association, and the specific formula (34) was as follows:


$$support\left(X\to Y\right)=\sigma \frac{\left(X\cup Y\right)}{N}$$

$$confidence\left(X\to Y\right)=\sigma \frac{\left(X\cup Y\right)}{\sigma \,\left(X\right)}$$

$$lift\left(X\to Y\right)=confidence\frac{\left(X\to Y\right)}{\sigma \,\left(Y\right)}$$

#### 2.5.7 Analysis of mediating effects

**FIGURE 2:**
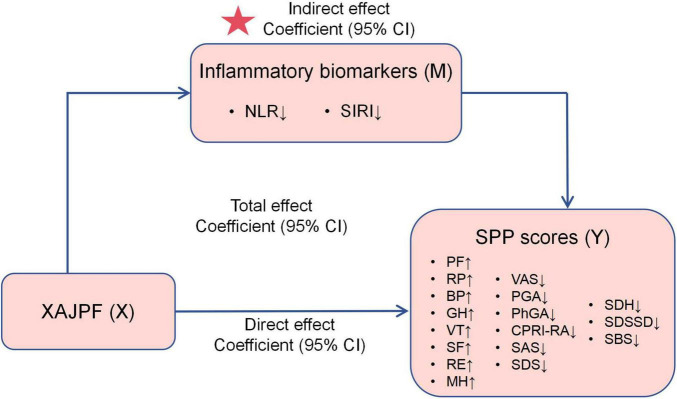
Schematic representation of the mediation effect. ↓ indicates a downward adjustment. ↑ indicates a upward adjustment. The star indicates an emphasized result.

The mediation analysis in this study was designed to elucidate a core mechanistic question: whether the improvement effect of XAJPF on SPP outcomes is partially mediated through the reduction of the systemic inflammatory indices of interest in this study (NLR and SIRI). We conducted a causal mediation analysis based on the methodology proposed by VanderWeele (35). The hypothesized causal model is shown in Figure 2, where XAJPF is the predictor variable (X), improvement in systemic inflammatory markers (NLR/SIRI) is the mediator (M), and improvement in SPP outcomes is the outcome variable (Y). The statistical significance of these estimates and their 95% confidence intervals were derived using non-parametric bootstrap sampling, a method that accounts for uncertainty in both the mediator and outcome models (36).

#### 2.5.8 Subgroup stratification analysis

In subgroup analyses, participants were stratified according to baseline characteristics, including sex (male/female), age (<60/ ≥ 60), BMI (<18.5/18.5–24/ ≥ 24), smoking (yes/no), drinking (yes/no), course of disease (<9/ ≥ 9), and CCI (<4/ ≥ 4), which were used as modifiers for replicating logistic regression models to assess the association between association between XAJPF and SPP outcomes. Also, we tested the interaction effect between XAJPF treatment and individual stratification factors.

#### 2.5.9 Sensitivity analysis

In addition, we performed sensitivity analyses to test the robustness of the results. To verify the generalizability of the protective association of XAJPF for SPP, we repeated the logistic regression modeling analyses in all 810 individuals, including baseline control participants. Consistent with the primary analyses, we also progressively corrected for all baseline variables and inflammation metrics in Model 2 (partially adjusted model) and Model 3 (fully adjusted model), respectively.

## 3 Results

### 3.1 Clinical characteristics of the study population

A total of 935 RA patients eligible for analysis were identified for the study. Overall, the median age was 58 years, with 160 (17.11%) males and 775 (82.89%) females, a median BMI of 22.10, a median disease duration of 9 years, and a median CCI of 4.00. Table 1 lists the baseline characteristics of the case and control groups, based on the median baseline NLR level (which was 2.88). The results showed significant differences between the two groups in terms of gender, age and BMI. A final study subset of 810 participants was generated in a 1:1 ratio according to the PSM principle, which adjusted for differences in demographic characteristics such as gender, age, BMI, smoking, and drinking, showing no statistically significant difference between the two groups (*P* > 0.05). Similarly, there were no significant differences between the two groups in terms of course of disease and CCI after matching. In particular, participants with higher baseline NLR were more inclined to have higher levels of SIRI, ESR, hs-CRP, RF, and CCP compared to the control group, and this trend of difference remained more significant after matching. In addition, patients in the case group had significantly worse SPP outcome scores than controls, as evidenced by lower RP and BP levels and higher VAS, PGA, PhGA, CPRI-RA, SDS, SDH, SDSSD, and SBS scores.

**TABLE 1:** Clinical characteristics of the study population by median baseline NLR before and after PSM.

Characteristic	Before PSM				After PSM			
	Overall (*n* = 935)	Controls (NLR<2.88, *n* = 467)	Cases (NLR ≥ 2.88, *n* = 468)	*P-*value	Overall (*n* = 810)	Controls (NLR<2.88, *n* = 405)	Cases (NLR ≥ 2.88, *n* = 405)	*P-* value
Gender, n(%)				<0.001				0.918
Male	160(17.11)	54(11.56)	106(22.65)		109(13.46)	54(13.33)	55(13.58)	
Female	775(82.89)	413(88.44)	362(77.35)		701(86.54)	351(86.67)	350(86.42)	
Age, year	58.00(16.00)	57.00(17.00)	60.00(17.00)	0.002	58.00(16.00)	57.00(17.00)	59.00(16.00)	0.314
Age, n(%)				0.001				0.104
<60	514(54.97)	283(60.60)	231(49.36)		453(55.93)	238(58.77)	215(53.09)	
≥60	421(45.03)	184(39.40)	237(50.64)		357(44.07)	167(41.23)	190(46.91)	
BMI, kg/m^2^	22.10(4.33)	22.27(4.08)	21.84(4.40)	0.005	22.06(4.27)	22.04(4.06)	22.15(4.42)	0.897
Smoking, n(%)	121(12.94)	46(9.85)	75(16.03)	0.260	91(11.23)	46(10.82)	45(11.11)	0.911
Drinking, n(%)	185(19.79)	82(17.56)	103(22.01)	0.088	148(18.27)	74(18.27)	74(18.27)	1.000
Course of disease, year	9.00(15.00)	10.00(14.00)	8.50(16.00)	0.392	10.00(15.23)	10.00(14.00)	9.00(16.00)	0.078
CCI	4.00(2.00)	4.00(2.00)	4.00(2.00)	0.004	4.00(2.00)	4.00(2.00)	4.00(2.00)	0.423
SIRI	1.18(1.15)	0.82(0.57)	1.87(1.33)	<0.001	1.18(1.12)	0.82(0.58)	1.84(1.30)	<0.001
ESR, mm/h	30.00(38.00)	25.00(32.00)	36.00(41.00)	<0.001	30.00(40.00)	25.00(33.00)	36.00(41.00)	<0.001
Hs-CRP, mg/L	14.08(33.73)	6.48(17.95)	25.07(47.37)	<0.001	13.54(32.97)	6.55(19.07)	22.72(44.92)	<0.001
RF, KIU/L	107.10(213.50)	94.30(183.70)	133.75(264.68)	0.001	102.90(211.83)	85.60(184.30)	133.00(264.60)	0.001
CCP, U/mL	95.30(252.50)	82.00(225.20)	102.00(281.27)	0.031	90.55(254.33)	76.40(230.15)	98.50(279.45)	0.039
PF	30.00(20.00)	30.00(20.00)	25.00(18.75)	0.001	30.00(20.00)	30.00(20.00)	25.00(15.00)	0.054
RP	0.00(25.00)	0.00(25.00)	0.00(25.00)	0.005	0.00(25.00)	0.00(25.00)	0.00(25.00)	<0.001
BP	31.00(10.01)	32.00(10.01)	31.00(19.00)	0.004	31.00(10.01)	32.00(11.01)	31.00(19.00)	0.002
GH	30.00(15.00)	30.00(15.00)	30.00(15.00)	0.564	30.00(15.00)	30.00(15.00)	30.00(15.00)	0.600
VT	40.00(20.00)	40.00(20.00)	40.00(20.00)	0.281	40.00(20.00)	40.00(20.00)	40.00(20.00)	0.440
SF	50.00(25.00)	50.00(25.00)	50.00(25.00)	0.337	50.00(25.00)	50.00(25.00)	50.00(25.00)	0.869
RE	33.33(33.33)	33.33(33.33)	33.33(33.33)	0.433	33.33(33.33)	0.00(33.33)	33.33(33.33)	0.352
MH	44.00(16.00)	44.00(16.00)	44.00(16.00)	0.381	44.00(16.00)	44.00(16.00)	44.00(16.00)	0.197
VAS, cm	6.00(1.80)	5.50(1.30)	6.00(1.70)	<0.001	6.00(1.80)	5.60(1.30)	6.00(1.60)	<0.001
PGA, cm	6.00(1.60)	5.50(1.30)	6.00(1.80)	<0.001	6.00(1.70)	5.60(1.30)	6.00(1.80)	<0.001
PhGA, cm	5.75(1.40)	5.40(1.00)	6.00(1.80)	<0.001	5.80(1.40)	5.50(1.00)	6.00(1.70)	<0.001
CPRI-RA	10.21(2.45)	9.99(2.35)	10.40(2.42)	<0.001	10.25(2.37)	10.01(2.28)	10.43(2.36)	<0.001
SAS	52.50(10.00)	52.50(10.00)	52.50(8.75)	0.525	52.50(10.00)	52.50(11.25)	52.50(10.00)	0.598
SDS	61.25(8.75)	60.00(10.00)	61.25(10.00)	0.036	61.25(8.75)	60.00(10.00)	61.25(9.38)	0.036
SDH	16.00(7.00)	15.00(6.00)	17.00(6.00)	<0.001	16.00(7.00)	16.00(7.00)	17.00(6.00)	<0.001
SDSSD	14.00(8.00)	13.00(8.00)	16.00(8.00)	<0.001	14.00(8.00)	14.00(9.00)	16.00(8.00)	0.002
SBS	6.00(4.00)	5.00(4.00)	6.00(4.00)	0.059	6.00(4.00)	5.00(4.00)	6.00(4.00)	0.032

Categorical variables were shown as n (percent, %). Continuous variables were shown as median (Interquartile range, IQR). *P*-values for differences between groups were derived using a Pearson’s Chi-squared test or Mann-Whitney *U*- test.

### 3.2 Associative effects of systemic inflammation indices and SPP outcomes

**FIGURE 3:**
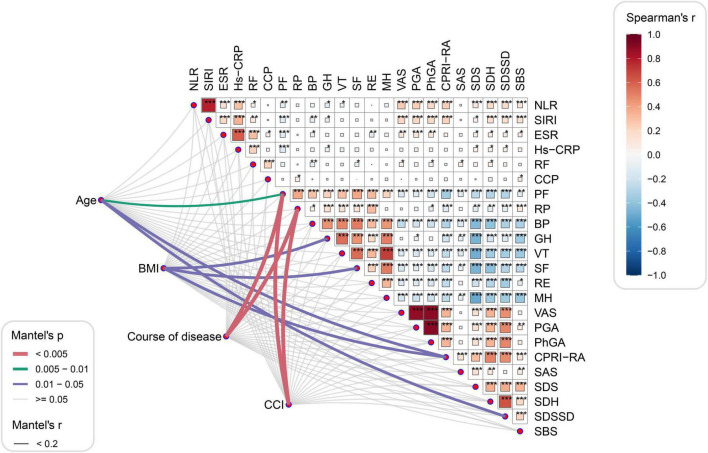
Correlation analysis between systemic inflammation index and SPP outcomes. *P<0.05, **P<0.01, and ***P<0.001.

Next, we focused on exploring potential associations between systemic inflammatory indices and SPP outcomes in the study population. The correlations of NLR, SIRI and baseline characteristics with SPP scores are illustrated in Figure 3. Spearman’s analysis showed that both NLR and SIRI were significantly positively correlated with ESR, hs-CRP, RF, VAS, PGA, PhGA, CPRI-RA, SDS, SDH, SDSSD and SBS levels and negatively correlated with PF and GH scores (Supplementary Table 2). In addition, NLR was also negatively correlated with VT and SIRI with BP, respectively. On the other hand, the Mantel test also found that age, BMI, disease duration, and CCI were the main influencing factors for PF, RP, GH, SF, CPRI-RA, and SDSSD scores (Supplementary Table 3).

**FIGURE 4:**
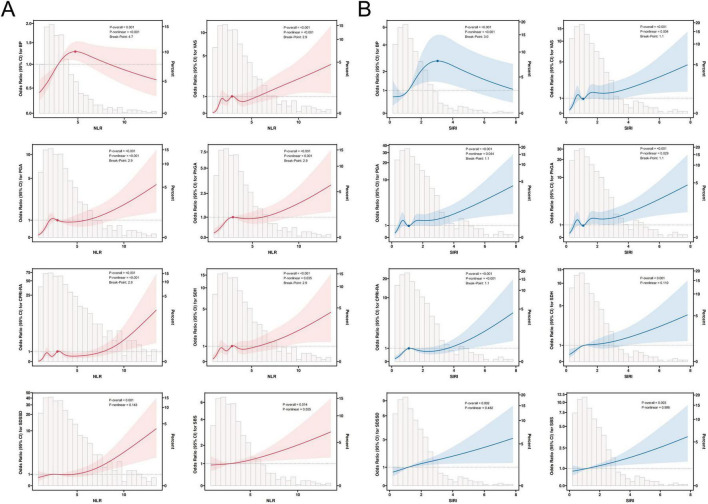
Restricted cubic spline regression analysis of NLR **(A)**, SIRI **(B)**, and SPP outcomes. The solid line in the figure indicates the OR and the shaded shapes indicate the 95% CI.

In Figure 4, we further plotted restricted cubic spline curves for visual modeling of the relationship between NLR and SIRI levels and the variance of SPP scores in each dimension. As a result, it was observed that there was a significant nonlinear association between NLR and BP, VAS, PGA, PhGA, CPRI-RA, and SDH scores (*P*-overall < 0.001, *P*-nonlinear < 0.05). Specifically, when NLR > 2.9, the risk of deterioration (OR) for the aforementioned scores significantly increased with rising NLR; interestingly, for BP scores, the risk showed a decreasing trend after NLR > 4.7. Additionally, NLR exhibited a linear correlation with the risk of SDSSD and SBS deterioration (*P*-overall < 0.05, *P*-nonlinear > 0.05). Similarly, SIRI exhibited nonlinear associations with BP, VAS, PGA, PhGA, and CPRI-RA scores (*P*-overall < 0.001, *P*-nonlinear < 0.05). SIRI > 1.1 marked a significant inflection point for risk escalation; conversely, when SIRI > 3.0, the risk of BP deterioration decreased. SIRI exhibited a linear correlation with the risk of SDH, SDSSD, and SBS deterioration (*P*-overall < 0.01, *P*-nonlinear > 0.05).

### 3.3 Outcome metrics for the case population with and without XAJPF

**FIGURE 5:**
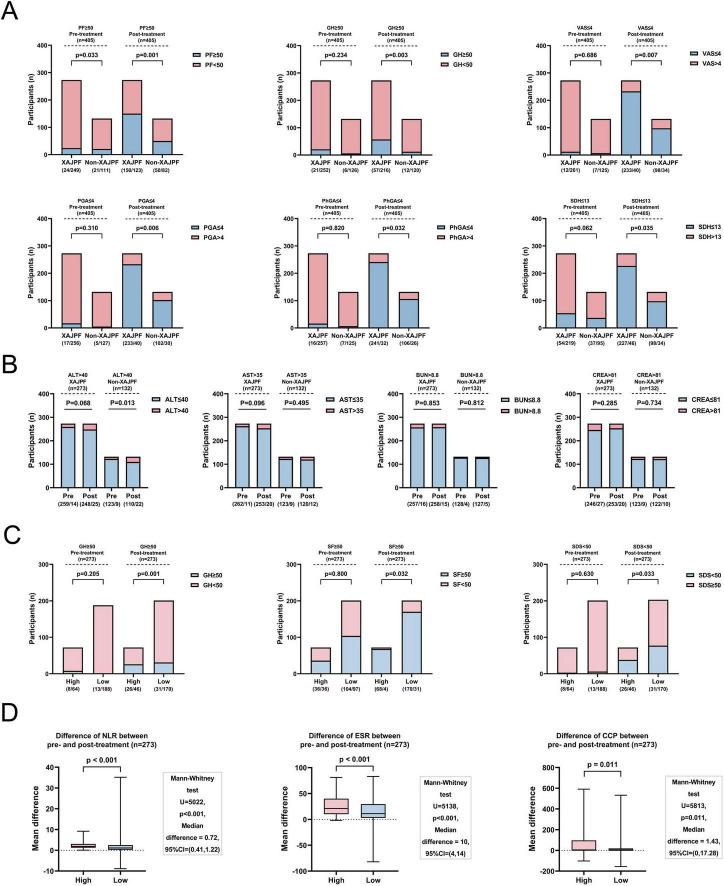
Outcome metrics for the XAJPF and Non-XAJPF groups, and for the high- and low-exposure groups of the population. **(A)** Proportion of patients with improved SPP outcome scores before and after treatment in the XAJPF and non-XAJPF groups. **(B)** Comparison of the proportion of patients with abnormal liver and kidney function indicators before and after treatment in the XAJPF and non-XAJPF groups. **(C)** Proportion of patients with improved SPP outcome scores before and after treatment in the high- and low-exposure groups. **(D)** Comparison of the mean differences in systemic inflammation outcome metrics between the pre- and post-treatment high and low-exposure groups.

A total of 273 (67.41%) participants in the case population were treated with XAJPF during the study period, and 132 (32.59%) participants were assigned to the non-XAJPF group (basic treatment only). Supplementary Table 4 shows that all baseline characteristics were largely comparable between the two groups (all, *P*> 0.05). The analysis of the primary outcome showed that XAJPF treatment had a more significant advantage than non-XAJPF treatment in improving the multidimensional aspects of patients’ overall functioning, symptoms, psychology, and TCM evidence (all, *P*<0.05), as reflected by the degree of elevation of PF, GH, VT, SF, RE, and MH, as well as VAS, PGA, PhGA, CPRI-RA, SAS, SDS, SDH, SDSSD, and SBS reduction levels (Table 2). As for RP and BP improvement, no statistically significant difference was observed between the two groups. In the analysis of the population of 405 cases, we also observed a higher percentage of improvement in PF, GH, VAS, PGA, PhGA, and SDH scores at the endpoints, defined primarily as a return of the scores to within the normal range or to the top 25% of the baseline scores (Figure 5A). Secondary outcome analysis showed that the XAJPF group demonstrated superior efficacy in reducing NLR, SIRI, ESR, and RF levels compared to the non-XAJPF group (all *P*< 0.01). Regarding the safety assessment, only the non-XAJPF group showed a mild increase in the proportion of abnormal ALT levels after treatment (*P* <0.05), while no other statistically significant differences were observed. This indicates that XAJPF treatment did not exhibit significant hepatorenal toxicity.

**TABLE 2:** Outcome metrics for the XAJPF-using and non-XAJPF-using populations in the case population.

Outcome indicators	Non-XAJPF group (*n* = 132)		XAJPF group (*n* = 273)		*P*-value
	Pre-treatment	Post-treatment	Pre-treatment	Post-treatment	
**SPP outcomes**					
PF	30.00(20.00)	40.00(25.00)*	25.00(20.00)	50.00(30.00)*	<0.001
RP	0.00(25.00)	50.00(25.00)*	0.00(25.00)	50.00(75.00)*	0.848
BP	31.00(10.01)	52.00(21.00)*	31.00(19.00)	52.00(32.00)*	0.242
GH	30.00(15.00)	35.00(20.00)*	30.00(15.00)	40.00(15.00)*	0.030
VT	40.00(15.00)	55.00(15.00)*	40.00(20.00)	60.00(15.00)*	0.018
SF	50.00(25.00)	62.50(25.00)*	50.00(25.00)	62.50(25.00)*	0.010
RE	33.33(33.33)	66.66(33.34)*	33.33(33.33)	66.66(83.50)*	0.046
MH	44.00(16.00)	56.00(16.00)*	44.00(20.00)	60.00(16.00)*	0.002
VAS, cm	6.00(1.48)	2.15(2.28)*	6.20(1.65)	2.10(1.80)*	0.001
PGA, cm	6.00(1.38)	2.20(2.10)*	6.10(1.90)	2.10(1.90)*	0.004
PhGA, cm	5.65(1.30)	2.00(2.18)*	6.00(1.80)	2.00(1.60)*	0.001
CPRI-RA	10.32(2.30)	4.63(3.04)*	10.48(2.45)	3.97(3.33)*	0.009
SAS	53.75(8.44)	46.25(8.75)*	51.25(10.00)	45.00(13.75)*	0.495
SDS	58.75(6.25)	52.50(7.50)*	62.50(9.38)	50.00(7.50)*	<0.001
SDH	16.00(6.00)	7.00(9.00)*	17.00(7.00)	6.00(7.00)*	<0.001
SDSSD	14.00(6.00)	7.00(5.00)*	16.00(8.00)	6.00(7.00)*	0.005
SBS	6.00(4.00)	4.00(2.00)*	6.00(4.00)	3.00(3.00)*	0.012
**Systemic inflammation outcomes**					
NLR	4.41(2.83)	3.84(2.56)*	4.08(1.87)	2.92(1.74)*	0.004
SIRI	1.80(1.31)	1.73(1.80)	1.86(1.29)	1.37(1.60)*	0.001
ESR, mm/h	30.00(37.00)	15.00(22.75)*	38.00(40.50)	17.00(25.50)*	0.007
Hs-CRP, mg/L	18.20(34.68)	2.61(8.89)*	26.73(44.48)	3.77(15.95)*	0.917
RF, KIU/L	123.25(222.05)	119.90(284.80)	138.00(265.60)	108.40(258.25)*	0.005
CCP, U/mL	98.68(243.96)	82.50(220.80)*	98.50(302.45)	78.00(240.05)*	0.841

Continuous variables were shown as median (Interquartile range, IQR). *P*-values for the within-group differences were performed by Wilcoxon Signed Rank test and annotated with * representing statistical significance. *P*-values for differences between groups were derived using the Mann-Whitney *U*-test.

In addition, the XAJPF group was further categorized into a low-exposure group (201 patients) and a high-exposure group (72 patients) based on the type of drug use. Supplementary Table 5 mainly showed that there was no statistical difference between the two groups in terms of demographic characteristics, disease duration, and comorbidities (all, *P*> 0.05). In fact, a higher proportion of GH, SF, and SDS scores improved in the high-exposure group, i.e., HQC combined with XFC, compared with the low-exposure group (HQC only or XFC only) (Figure 5B). In addition, the comparison of HQC combined with XFC and HQC alone or XFC alone showed that the combination therapy was more efficacious in reducing the levels of NLR, ESR, and CCP (all *P*< 0.05) (Figure 5C).

### 3.4 Association of XAJPF use with risk of adverse SPP outcomes in RA patients

**FIGURE 6:**
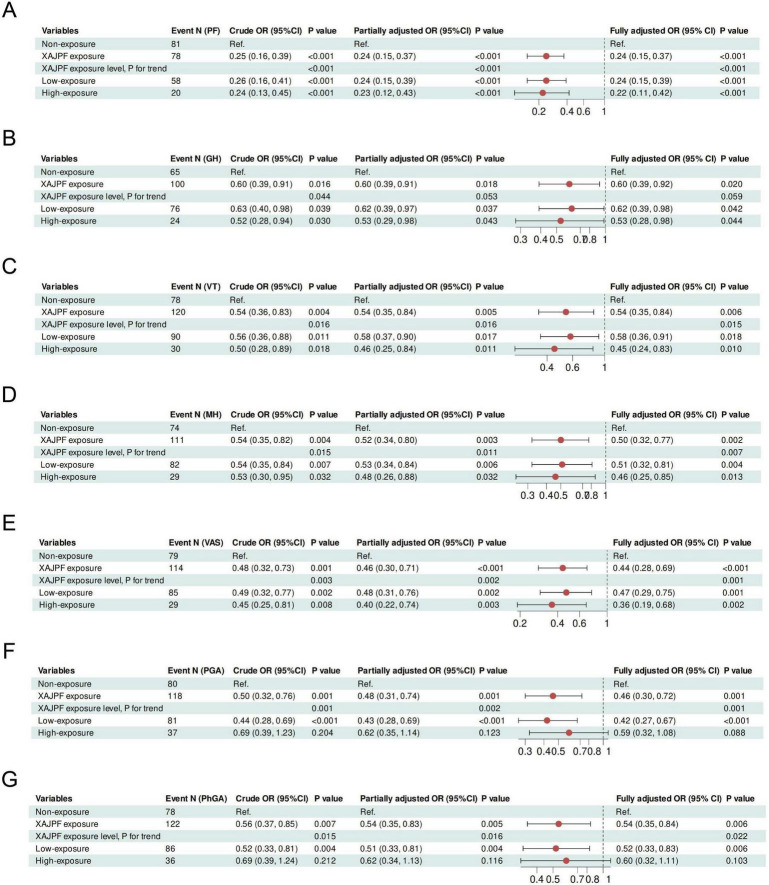
Association of XAJPF use with risk of adverse SPP outcomes in RA patients, including PF **(A)**, GH **(B)**, VT **(C)**, MH **(D)**, VAS **(E)**, PGA **(F)**, and PhGA **(G)**. Crude OR: unadjusted; Partially adjusted OR: adjusted for gender, age, BMI, smoking, drinking, course of disease, and CCI; Fully adjusted OR: adjusted for the terms in the partially adjusted model and for all baseline inflammatory indicators including NLR, SIRI, ESR, hs-CRP, RF, and CCP.

Next, we developed univariate and stepwise-adjusted logistic regression models to quantify the effect of XAJPF use on the risk of worsening SPP (Figure 6 and Supplementary Figure 1). In the initial crude model, compared with those who never used XAJPF, those with a history of XAJPF use were 75, 40, 46, 46, 46, 52, 51, and 51% less likely to have a worsening of PF, GH, VT, MH, VAS, PGA, PhG, CPRI-RA, SDS, SDH, and SDSSD, in that order, 44, 41, 55, 67, and 45% (all OR < 1, *p*< 0.05), suggesting that XAJPF is a key protective factor against adverse SPP outcomes in RA patients. Second, exposure was introduced as an ordinal variable, where the Non-XAJPF group served as the reference group. The results showed that in terms of PF, GH, VT, MH, VAS, and SDSSD scores, the risk of deterioration was significantly lower in highly exposed participants, and the risk index was consistently lower than that of the low-exposure group, implying a negative exposure-response correlation. Further insights from progressively adjusted logistic regression models emphasized the relationship between XAJPF and the defined risk of developing adverse SPP outcomes. We found that after final adjustment for all registry parameters, such as age, sex, BMI, disease duration, smoking, alcohol consumption, and various baseline inflammatory markers, the risk of worsening SPP was reduced by half in the XAJPF group. A similar trend was observed after stratification according to XAJPF. Notably, the risk of developing PF, GH, VT, MH, VAS, CPRI-RA, SDS, SDH, and SDSSD was significantly reduced with increasing exposure categories and was independent of all confounding parameters involved in the final adjusted model. Overall, use of XAJPF was an independent protective factor for the risk of adverse SPP outcomes, and this exposure-response relationship persisted in the cohort.

### 3.5 Combined association of XAJPF use categories with composite outcome improvement in RA patients

**FIGURE 7:**
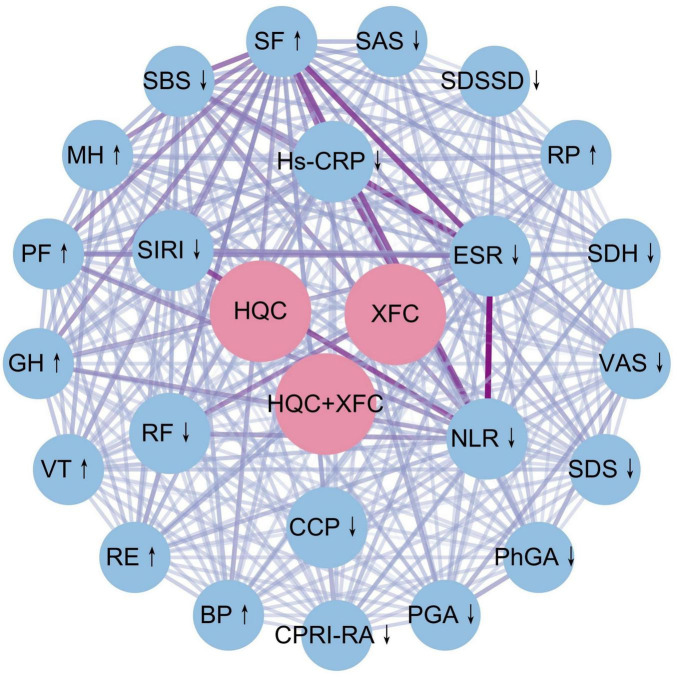
Complex network diagrams using combinatorial associations between XAJPF and improved outcome metrics in RA patients. ↓ indicates a downward adjustment. ↑ indicates a upward adjustment.

To further clarify the relationship between the type of XAJPF use and the improvement of outcome indicators, an association rule analysis based on Apriori module was performed to obtain the high-frequency drug-indicator combinations and their effect strengths. Figure 7 demonstrates, based on the complex network model, that XAJPF has a strong association with the improvement of immune-inflammatory indicators and SPP scores in each dimension in RA patients. Setting the type of exposure as the antecedent and the improvement of immune-inflammatory indicators and SPP scores as the posterior, the results in Table 3 showed that the application of HQC alone was strongly associated with the reduction of ESR, NLR, and SIRI indicators, and with the increase of PF and GH scores; and the use of XFC alone was strongly associated with the improvement of hs-CRP, NLR, SBS, PGA, SAS, RF, SIRI, PhGA, SDH, VAS, SDS, and CPRI-RA levels were associated with decreased levels of SF, RE, PF, MH, and BP, and with increased levels of SF, RE, PF, MH, and BP; furthermore, the combination of HQC and XFC was strongly associated with decreased levels of NLR, ESR, SIRI, RF, and SDSSD, and strongly associated with increased PF and GH scores. All of the above associations had a support level greater than 15%, a confidence level greater than 60%, and an elevation level greater than 1.

**TABLE 3:** Combined association between XAJPF and improvement of outcome indicators in RA patients.

Items (The antecedent ⇒ the consequent)		Support (%)	Confidence (%)	Lift
HQC	ESR↓	29.38	82.35	1.029
HQC	NLR↓	29.38	81.51	1.028
HQC	PF↑	29.38	72.27	1.190
HQC	GH↑	29.38	66.39	1.120
HQC	SIRI↓	29.38	63.87	1.010
XFC	Hs-CRP↓	20.25	82.93	1.146
XFC	SF↑	20.25	82.93	1.083
XFC	NLR↓	20.25	80.49	1.016
XFC	SBS↓	20.25	78.05	1.225
XFC	PGA↓	20.25	71.95	1.408
XFC	RE↑	20.25	70.73	1.262
XFC	SAS↓	20.25	69.51	1.316
XFC	RF↓	20.25	69.51	1.126
XFC	PF↑	20.25	69.51	1.144
XFC	SIRI↓	20.25	67.07	1.061
XFC	PhGA↓	20.25	65.85	1.301
XFC	SDH↓	20.25	64.63	1.271
XFC	VAS↓	20.25	64.63	1.235
XFC	MH↑	20.25	64.63	1.190
XFC	SDS↓	20.25	63.41	1.259
XFC	CPRI-RA↓	20.25	63.41	1.217
XFC	BP↑	20.25	60.98	1.170
HQC++XFC	NLR↓	17.78	100.00	1.262
HQC+XFC	ESR↓	17.78	97.22	1.215
HQC+XFC	SIRI↓	17.78	77.78	1.230
HQC+XFC	PF↑	17.78	72.22	1.189
HQC+XFC	RF↓	17.78	66.67	1.080
HQC+XFC	GH↑	17.78	66.67	1.125
HQC+XFC	SDSSD↓	17.78	61.11	1.184

↓ Indicates a downward adjustment. ↑ indicates a upward adjustment.

### 3.6 Mediating effects of systemic inflammation indices on the association between XAJPF and improved SPP outcomes

**FIGURE 8:**
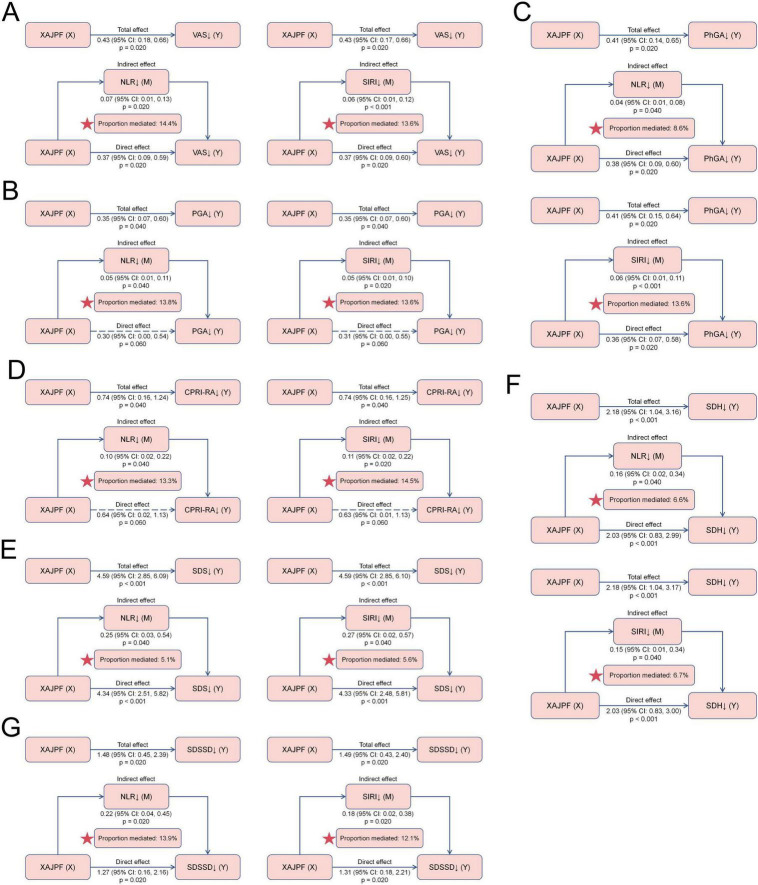
Mediating effects of systemic inflammation indices on the association between XAJPF and improved SPP outcomes, including VAS **(A)**, PGA **(B)**, PhGA **(C)**, CPRI-RA **(D)**, SDS **(E)**, SDH **(F)**, and SDSSD **(G)**. ↓ indicates a downward adjustment. The star indicates an emphasized result.

In addition, we tested NLR and SIRI as potential mediators of the association between XAJPF and improved SPP outcomes. Mediation analyses showed that NLR and SIRI both mediated the relationship between XAJPF and SPP outcomes to some extent (Figure 8). NLR and SIRI mediated a greater proportion of indirect effects in the following relationships, respectively. For VAS, the proportion of indirect effects mediated by NLR and SIRI was 14.4 and 13.6%, respectively. Regarding PhGA, the proportion of indirect effects mediated by SIRI was relatively higher at 13.6%. Interestingly, the NLR- and SIRI-mediated indirect effects between XAJPF and improvement in PGA and CPRI-RA scores were significant, with specific proportions of 13.8, 13.6, 13.3, and 14.5%, respectively, whereas none of the direct effects were significant, suggesting that there may be a fully mediated effect. In addition, NLR and SIRI partially mediated the relationship between XAJPF and improvement in SDSSD scores, with mediating proportions of 13.9 and 12.1%, respectively.

### 3.7 Subcomponent stratification and interaction effects of XAJPF use with SPP outcomes in RA patients

**FIGURE 9:**
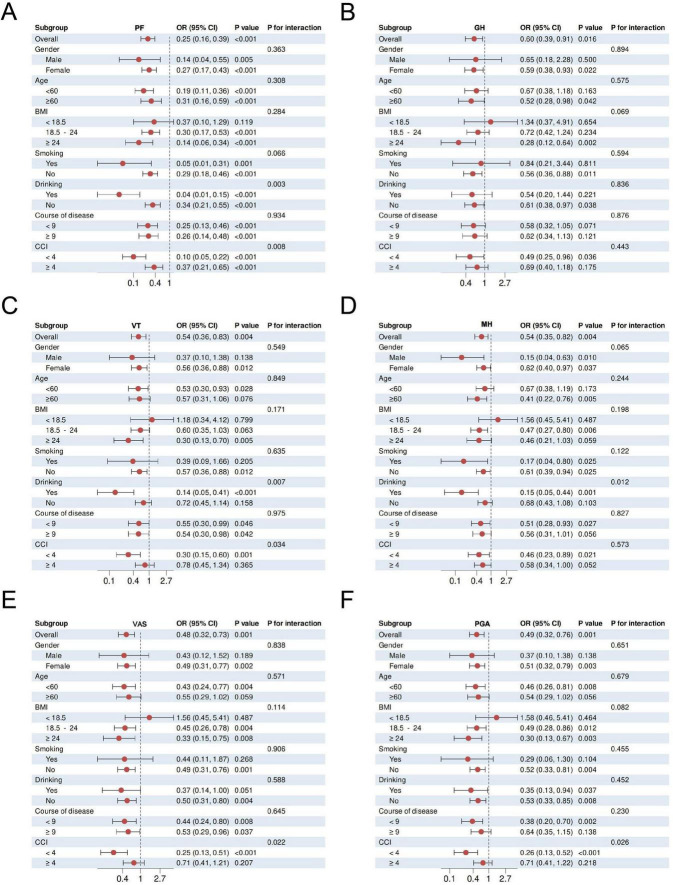
Subcomponent stratification and interaction effects of XAJPF with SPP outcomes in RA patients, including PF **(A)**, GH **(B)**, VT **(C)**, MH **(D)**, VAS **(E)**, and PGA **(F)**.

To further evaluate the interaction effect of the protective effect of XAJPF use on SPP outcomes in RA patients, we conducted subgroup stratification analyses based on gender, age, BMI, smoking, drinking, course of disease, and CCI, respectively. Stratified analyses showed that the protective association between XAJPF and SPP outcomes was broadly consistent across stratification factors (Figure 9 and Supplementary Figure 2). Consistent protective effects of XAJPF against VAS, PGA, and PhGA were observed in most strata, occurring predominantly in females, patients < 60 years of age, with a BMI of 18.5–24 or ≥ 24, non-smokers, non-drinkers, with course of disease < 9 years, and with a CCI < 4. The protective effect of XJTP against CPRI-RA was more frequently observed in females, age < 60 years, BMI 18.5–24 or ≥ 24, and non-smokers. The protective effect of XAJPF on SDSSD was more likely to occur in females, age < 60 years, BMI 18.5–24 or ≥ 24, non-smokers, drinkers, course of disease ≥ 9 years, and CCI <4. Significant associations between XAJPF and GH were more likely to occur in women, age < 60 years, BMI ≥ 24, non-smokers, drinkers, and CCI < 4. Significant correlations between XAJPF and VT were concentrated in women, age < 60 years, BMI ≥ 24, non-smokers, non-drinkers, and CCI < 4. In addition, the protective effect of XAJPF against SDSSD occurred more frequently in individuals who were female, aged < 60 years, BMI 18.5–24 or ≥ 24, non-smokers, and CCI <4. As for the protective associations between XAJPF and PF, SDH, it was beneficial in all stratified individuals except for participants with BMI <18.5. In particular, in the stratified analyses of logistic regression models, we observed that CCI could influence the relationship between XAJPF and VAS, PGA, PhGA, VT, and SDS, with BMI predominantly acting on PhGA, CPRI-RA, and SDS (*p*< 0.05 for interaction). In addition, we identified drinking as an interaction factor through the association between XAJPF and SDH, PF, and MH (interaction *P*< 0.05).

### 3.8 Sensitivity analysis

We performed sensitivity analyses, which were primarily based on constructing models in the full cohort population of 810 cases after matching (Supplementary Table 6). Binary logistic regression analyses showed positive and robust protective associations between XAJPF use and the risk of deterioration in PF, GH, VT, MH, CPRI-RA, SDS, SDH, and SDSSD. Both low and high exposure levels were associated with a reduced relative risk of deterioration in PF, VT, CPRI-RA, SDS, and SDH compared with the non-exposed group. In addition, we made additional adjustments for baseline characteristics separately and further corrected for baseline levels of inflammatory metrics on the basis of this model. The results were broadly consistent with the primary analysis, which provides ample evidence of the robustness of our results.

## 4 Discussion

RA is a multi-system inflammatory disease involving bone, cartilage and synovial tissues, characterized by progressive joint structural destruction, dysfunction and multiple complications, with high morbidity and risk of disability (37). In the pathological process of RA, abnormal infiltration of immune cells and excessive release of inflammatory mediators constitute the core driving mechanisms (38), which not only exacerbate the tissue damage, but also significantly affect the patient’s self-perception and clinical prognosis. Currently, the goal of RA treatment has shifted from inflammation control to “treat-to-target,” i.e., to achieve both biological remission and functional improvement, which may be more beneficial to RA patients. However, due to the low level of evidence and single dimension of efficacy in previous studies, it is difficult to fully reflect the overall efficacy of XAJPF. Our findings suggest that XAJPF treatment greatly improves the systemic inflammation and SPP composite outcomes in RA patients and that the protective association between the risk of adverse SPP is partially mediated by the systemic inflammation index.

Assessing the severity of disease activity in RA patients has long remained a challenging topic. Against this backdrop, this study introduces the SPP scale. This instrument integrates elements of TCM syndromes, such as damp-heat syndrome, spleen deficiency with dampness syndrome, and blood stasis syndrome, into a comprehensive assessment of overall function, physical pain, and psychological impairment. It aligns closely with the ICHOM framework for standardized outcome assessment in inflammatory arthritis. Compared to conventional indicators primarily reflecting platelet activity, the NLR and SIRI metrics examined in this study directly target the core effector immune cells of RA. In active RA patients, a high inflammatory state drives abnormal neutrophil proliferation through anti-apoptotic mechanisms and granulocyte colony-stimulating factor, forming a positive feedback inflammatory loop (39), while lymphopenia correlates with inflammatory chemotaxis-induced lymphocyte migration, infiltration, and accelerated apoptosis (40). Chandrashekara et al. (41) demonstrated that NLR exhibits comparable predictive value to CRP for VAS, swollen joint count, and SF-36 scores, with superior stability and minimal interference from certain cytokines. Similarly, in this study, subjects with elevated NLR generally exhibited higher ESR, Hs-CRP, RF, and anti-CCP antibody levels, along with poorer SPP scores. As another systemic inflammatory composite indicator, elevated SIRI correlates closely with monocytosis. The latter promotes osteoclast differentiation mediated by factors such as IL-26, contributing to RA bone erosion (42). Multiple studies suggest SIR predicts RA-associated interstitial lung disease and malignancy risk (43). Our correlation and RCS curve analyses further revealed that inflammation load exceeding specific thresholds (e.g., NLR > 2.9 or SIRI > 3.0) may accelerate the deterioration of SPP macro-level outcomes. Therefore, this study integrated a multidimensional framework for evaluating RA treatment efficacy, encompassing not only the biological basis of the disease and patient-reported outcomes but also highlighting the distinctive features of TCM syndromes. This approach facilitates the direct capture of the dynamic interaction between inflammation and perception in RA.

According to the traditional theory, hospitalized RA patients in the active stage mostly show signs of damp-heat paralysis caused by spleen deficiency. Consequently, HQC, composed of Scutellaria baicalensis, Gardenia jasminoides, Radix clematidis, Coicis semen, and Peach kernel, primarily targets the characteristics of damp-heat obstruction syndrome. XFC, serving as a foundational representative formula for spleen deficiency with dampness encumbrance syndrome, consists of Astragalus membranaceus, Coicis semen, Centipede, and Tripterygium wilfordii. Pharmacological studies revealed that baicalin from Scutellaria baicalensis can induce apoptosis in synovial monocytes by inhibiting JAK1/STAT3 signaling, thereby alleviating synovial inflammation and significantly improving the pressure pain threshold and arthritis scores in RA mice (44). Wang et al. (45) found that gardenoside downregulates SphK1 translocation mediated by the VEGFR2/PKC/ERK1/2 pathway and inhibits S1P/S1PR1 signaling activation, thereby reducing VEGF-stimulated angiogenesis in arthritic mice. Extracts of Radix clematidis may improve RA pathology by further blocking the circPTN/miR-145-5p/Wnt/β-catenin signaling axis through binding to FZD4 (46). Coicis semen has also been reported to reduce synovial angiogenesis in RA by inhibiting the HIF-1α/VEGF-A signaling pathway via SIRT1 (47). Peach kernel extract exhibits hematological improvement effects in addition to anti-arthritic properties (48). Previous studies also revealed that astragaloside IV modulates lncRNA LOC100912373 and miR-17-5p/PDK1 axis expression to inhibit synovial cell proliferation and pathological progression (49). Our study found that XAJPF treatment significantly reduced systemic inflammation indices and improved multidimensional SPP scores in the primary case cohort, demonstrating markedly superior efficacy compared to non-XAJPF-treated individuals. Furthermore, the safety profile of XAJPF warrants attention. Preclinical toxicity studies in rats demonstrated that continuous oral administration of XFC at various doses over 6 months did not induce abnormal changes in hematological parameters or vital organ tissues such as the liver and kidneys (50). This study aligns with prior large-scale cohort investigations and randomized controlled trials (12, 13), confirming that hepatic and renal function markers remain relatively stable following XAJPF treatment. Further insights from the multivariable-adjusted logistic regression model suggest XAJPF may represent an independent protective factor against the risk of defined adverse SPP outcomes. In summary, this study captures the advantages of XAJPF as TCM compounds for holistic regulation and integrated mind-body treatment, constituting a crucial supplement and refinement to prior pharmacological evidence.

TCM has been applied to the treatment of paralysis in China for thousands of years, and has developed a systematic theoretical framework and evidence-based clinical practice protocols. Our research highlights a pivotal finding through composite outcome and mediation analysis: compared with monotherapy, combined treatment with HQC and XFC demonstrates superior efficacy in reducing NLR, ESR, and CCP levels, improving the proportion of patients achieving higher GH, SF, and SDS scores, and lowering the risk of SPP. Previous studies indicate that HQC exerts anti-RA effects by inhibiting neutrophil extracellular trap production and inflammatory progression mediated by the p38MAPK signaling pathway through regulating circ0005732 expression (51). Li et al. (52) further proposed that HQC may activate the Wnt1/β-catenin signaling pathway to improve inflammatory responses and depressive-like behaviors in RA rats, revealing a novel mechanism of HQC in RA-SPP treatment. Similarly, XFC has been demonstrated to modulate the lncRNA DSCR9/RPLP2/PI3K/AKT axis to alleviate RA inflammation and hypercoagulability (53). Compared to these single-agent studies, this research directly validated the synergistic effects of combination therapy in dual immunological-psychological regulation within a large clinical cohort. Notably, NLR and SIRI mediated 14.4 and 13.6% of XAJPF’s effect on VAS score improvement, respectively, while 13.9% of SDSSD outcome improvement was driven by NLR reduction. In summary, our findings not only validate the proportion of systemic inflammation mediated within the framework of overall therapeutic efficacy assessment, but more significantly, reveal the remarkable regulatory capacity of the TCM compounds over the core immune network comprising neutrophils, lymphocytes and monocytes.

In addition, we observed partial population heterogeneity in the efficacy of XAJPF on SPP outcomes by subcomponent stratification. Female patients generally benefited more from XAJPF treatment compared to men, which may be related to differences in estrogen-regulated immune response (54). Patients with BMI ≥ 24 demonstrated more pronounced improvements in GH and VT scores, potentially because XAJPF may enhance efficacy by exerting anti-inflammatory effects through regulating lipid metabolism (55). Conversely, patients with CCI ≥ 4 exhibited diminished efficacy in VAS, PGA, PhGA, and SDS scores, reflecting the constraints imposed by multiple pathological burdens on the response to TCM. Additional sensitivity analyses further underscored the universality and robustness of XAJPF’s protective association with SPP. Collectively, these findings suggest that XAJPF suppresses systemic inflammatory responses and improves clinical SPP outcome scores in RA patients.

This pioneering work presents several important advantages. First, the study design was completed for the record based on the International Traditional Medicine Clinical Trial Registry Platform, which strictly followed the international norms and ensured the transparency and reproducibility of the methodology. Second, a composite outcome evaluation system was innovatively constructed, realizing the organic integration of international standard outcomes and TCM characteristic evidence by integrating objective biological indicators and subjective functional feedback. Third, the design of NLR pre-stratification and case-control was applied, relying on PSM to strictly control confounding factors, and the reliability of causal inference was greatly enhanced by sensitivity analysis. However, some noteworthy limitations must be recognized. First, our findings focused on evaluating the efficacy of TCM formulas, lacking specific analysis of other medication use (such as DMARDs usage records), which may introduce residual confounding bias. Second, data from a single-center source may exhibit selection bias, such as limited representativeness of the patient population and systematic errors in the implementation of inclusion/exclusion criteria, which could affect the generalizability of the results. Third, due to the limitations of inpatient follow-up, the observation period was confined to the interval between hospital admission and discharge, which restricted the systematic evaluation of the long-term efficacy and safety of XAJPF. In future studies, we will expand the sample size and conduct multicenter, prospective, long-term cohort follow-ups to comprehensively validate and extend the research findings.

## 5 Conclusion

This study demonstrated that XAJPF effectively improves systemic inflammation levels and multidimensional SPP scores in RA patients. Changes in systemic inflammatory markers NLR and SIRI are correlated with SPP outcome progression in RA patients and may mediate XAJPF’s protective effect on multidimensional SPP outcomes. These findings emphasize XAJPF’s unique advantage in improving systemic inflammation and SPP composite outcomes in RA patients, providing evidence-based decision support for the application of TCM in RA clinical management.

## Data Availability

The original contributions presented in this study are included in this article/Supplementary material, further inquiries can be directed to the corresponding author.
