# Supplementary material for: Multidimensional effects of the Xin’an Jianpi Tongbi Formula on self-perception of patients with rheumatoid arthritis: focusing on the mediating role of systemic inflammation index
Source: Front Med (Lausanne). 2026 Jan 30;13:1756862. doi: 10.3389/fmed.2026.1756862 (PMC12901419; doi:10.3389/fmed.2026.1756862)
Supplement: Supplementary file 1 [file Data_Sheet_1.docx]

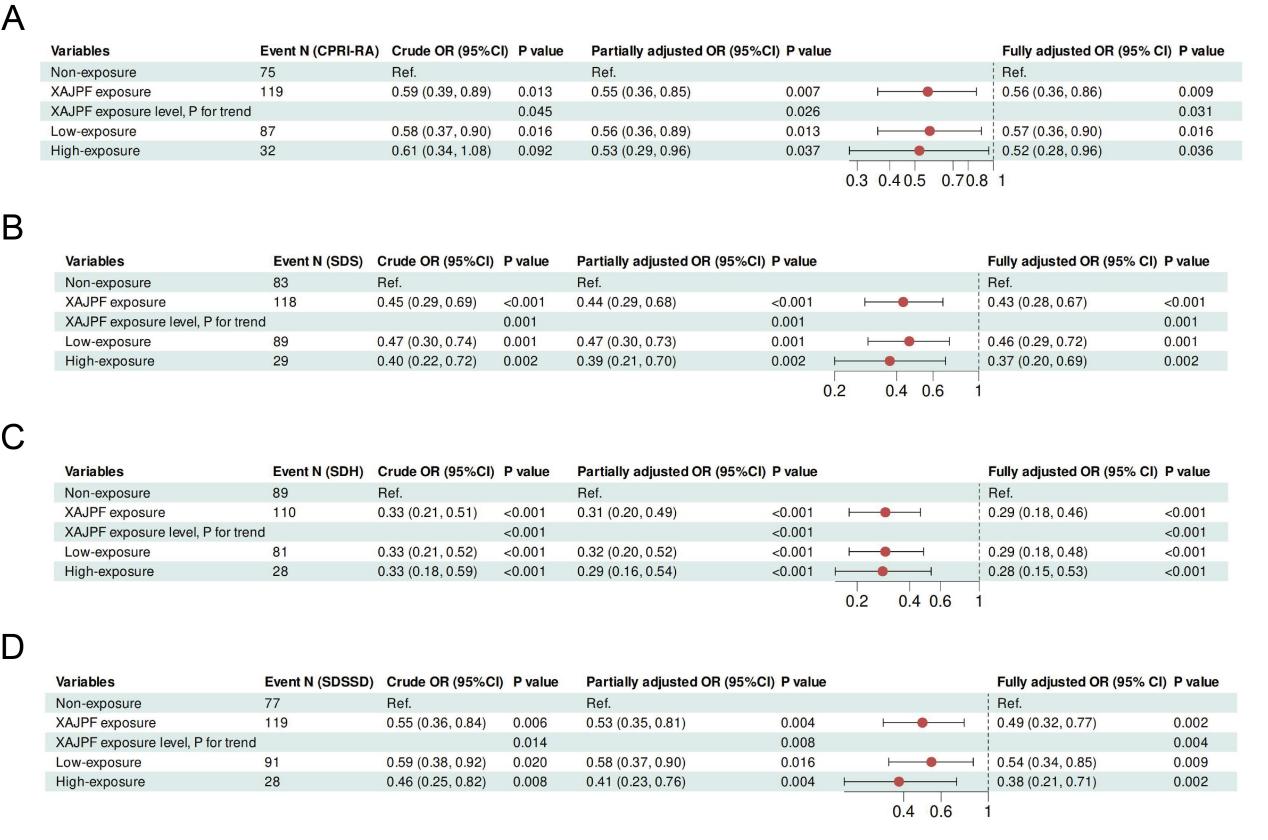


Supplementary Figure 1 Risk association of XAJPF use with adverse SPP outcomes in RA patients, including CPRI-RA (A), SDS (B), SDH (C) and SDSSD (D).

Notes: Crude OR: unadjusted; Partially adjusted OR: adjusted for gender, age, BMI, smoking, drinking, course of disease, and CCI; Fully adjusted OR: adjusted for the terms in the partially adjusted model and for all baseline inflammatory indicators including NLR, SIRI, ESR, hs-CRP, RF, and CCP.


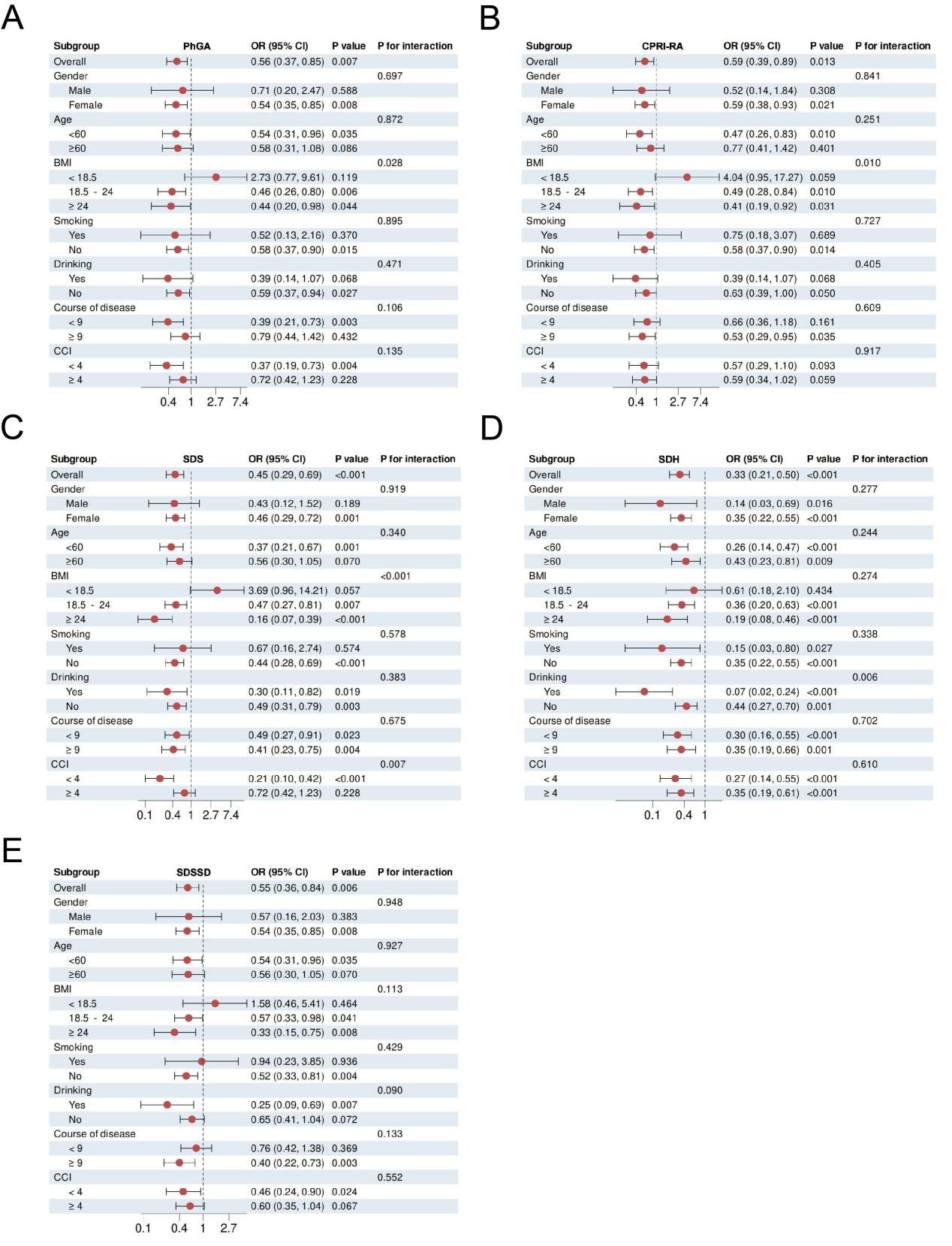


Supplementary Figure 2 Subcomponent stratification and interaction effects of XAJPF with SPP outcomes in RA patients, including PhGA (A), CPRI-RA (B), SDS (C), SDH (D), and SDSSD (E).
