# Supplementary material for: Multidimensional effects of the Xin’an Jianpi Tongbi Formula on self-perception of patients with rheumatoid arthritis: focusing on the mediating role of systemic inflammation index
Source: Front Med (Lausanne). 2026 Jan 30;13:1756862. doi: 10.3389/fmed.2026.1756862 (PMC12901419; doi:10.3389/fmed.2026.1756862)
Supplement: Supplementary file 2 [file Data_Sheet_2.docx]

Supplementary Table 1 Chemical Constituents and Pharmacological Properties of Active Components in XAJPF

| Capsule | Active Component | Source Composition | Chemical Class | Main Pharmacological Actions | Reference |
| --- | --- | --- | --- | --- | --- |
| HQC | Baicalin | Scutellaria baicalensis | Flavone glycoside | Anti-inflammatory, Antioxidant, Immunomodulation, Antibacterial | (9, 10) |
|  | Baicalein | Scutellaria baicalensis | Flavone aglycone | Anti-inflammatory, Antitumor, Neuroprotective, Antioxidant |  |
|  | Geniposide | Gardenia jasminoides | Iridoid glycoside | Anti-inflammatory, Hepatoprotective, Neuroprotective, Antidepressant |  |
|  | Oleanolic acid | Radix clematidis | Pentacyclic triterpenoid acid | Anti-inflammatory, Hepatoprotective, Antidiabetic, Immunomodulation |  |
|  | Luteolin | Radix clematidis | Flavonoid | Anti-inflammatory, Antioxidant, Anti-allergic, Anticancer |  |
|  | Coixol | Coicis Semen | Benzoxazinone | Sedative, Analgesic, Anti-inflammatory, Antitumor |  |
|  | Amygdalin | Peach kernel | Cyanogenic glycoside | Antitussive, Anti-inflammatory, Potential antitumor |  |
| XFC | Calycosin-7-glucoside | Astragalus membranaceus | Isoflavone glycoside | Qi-invigorating, Immunoregulation, Antioxidant, Cardioprotective | (11) |
|  | Calycosin | Astragalus membranaceus | Isoflavone | Anti-inflammatory, Antioxidant, Angiogenic, Neuroprotective |  |
|  | Formononetin | Astragalus membranaceus | Isoflavone | Estrogenic, Anti-inflammatory, Antioxidant, Osteogenic |  |

Supplementary Table 2 Correlation between NLR, SIRI and laboratory indicators, SPP outcomes

|  | | NLR | | SIRI | |
| --- | --- | --- | --- | --- | --- |
|  |  | r | p | r | p |
| Laboratory indicators | ESR | 0.156 | <0.001 | 0.205 | <0.001 |
|  | Hs-CRP | 0.284 | <0.001 | 0.348 | <0.001 |
|  | RF | 0.072 | 0.040 | 0.113 | 0.001 |
|  | CCP | -0.007 | 0.851 | 0.008 | 0.822 |
| SPP outcomes | PF | -0.105 | 0.003 | -0.122 | 0.001 |
|  | RP | 0.035 | 0.322 | 0.031 | 0.374 |
|  | BP | -0.065 | 0.066 | -0.097 | 0.006 |
|  | GH | -0.089 | 0.011 | -0.080 | 0.023 |
|  | VT | -0.069 | 0.049 | -0.033 | 0.348 |
|  | SF | -0.060 | 0.086 | -0.029 | 0.415 |
|  | RE | -0.001 | 0.984 | -0.021 | 0.558 |
|  | MH | -0.060 | 0.088 | -0.021 | 0.546 |
|  | VAS | 0.253 | <0.001 | 0.211 | <0.001 |
|  | PGA | 0.244 | <0.001 | 0.210 | <0.001 |
|  | PhGA | 0.196 | <0.001 | 0.200 | <0.001 |
|  | CPRI-RA | 0.241 | <0.001 | 0.226 | <0.001 |
|  | SAS | -0.015 | 0.679 | 0.010 | 0.779 |
|  | SDS | 0.119 | 0.001 | 0.085 | 0.015 |
|  | SDH | 0.186 | <0.001 | 0.158 | <0.001 |
|  | SDSSD | 0.182 | <0.001 | 0.160 | <0.001 |
|  | SBS | 0.124 | <0.001 | 0.153 | <0.001 |

Supplementary Table 3 Correlation between baseline characteristics and systemic inflammation index, SPP outcomes

|  | | Baseline characteristics | | | | | | | |
| --- | --- | --- | --- | --- | --- | --- | --- | --- | --- |
|  |  | Age | | BMI | | Course of disease | | CCI | |
|  |  | r | p | r | p | r | p | r | p |
| Immune-inflammatory indicators | NLR | 0.003 | 0.399 | 0.026 | 0.104 | -0.030 | 0.951 | -0.03 | 0.951 |
|  | SIRI | -0.011 | 0.646 | 0.019 | 0.159 | -0.029 | 0.925 | -0.029 | 0.953 |
|  | ESR | 0.024 | 0.090 | -0.002 | 0.512 | 0.011 | 0.222 | 0.011 | 0.230 |
|  | Hs-CRP | -0.015 | 0.733 | 0.033 | 0.078 | -0.018 | 0.824 | -0.018 | 0.824 |
|  | RF | 0.012 | 0.309 | 0.003 | 0.433 | 0.001 | 0.461 | 0.001 | 0.461 |
|  | CCP | -0.019 | 0.817 | 0.024 | 0.110 | 0.028 | 0.058 | 0.028 | 0.058 |
| SPP outcomes | PF | 0.048 | 0.008 | 0.011 | 0.266 | 0.064 | 0.001 | 0.064 | 0.001 |
|  | RP | -0.023 | 0.904 | -0.034 | 0.979 | 0.072 | 0.002 | 0.072 | 0.002 |
|  | BP | -0.011 | 0.737 | 0.018 | 0.195 | 0.005 | 0.357 | 0.005 | 0.357 |
|  | GH | -0.001 | 0.500 | 0.032 | 0.045 | 0.022 | 0.084 | 0.022 | 0.084 |
|  | VT | 0.015 | 0.178 | 0.003 | 0.411 | 0.014 | 0.150 | 0.014 | 0.150 |
|  | SF | 0.000 | 0.473 | 0.038 | 0.012 | -0.021 | 0.928 | -0.021 | 0.936 |
|  | RE | 0.004 | 0.378 | 0.000 | 0.446 | 0.017 | 0.100 | 0.017 | 0.100 |
|  | MH | 0.005 | 0.389 | -0.015 | 0.828 | -0.017 | 0.875 | -0.017 | 0.875 |
|  | VAS | -0.010 | 0.720 | -0.002 | 0.542 | -0.004 | 0.592 | -0.004 | 0.592 |
|  | PGA | -0.017 | 0.841 | -0.003 | 0.545 | 0.003 | 0.408 | 0.003 | 0.408 |
|  | PhGA | -0.026 | 0.941 | -0.007 | 0.650 | 0.015 | 0.168 | 0.015 | 0.168 |
|  | CPRI-RA | 0.038 | 0.036 | 0.040 | 0.033 | 0.007 | 0.334 | 0.007 | 0.354 |
|  | SAS | 0.014 | 0.263 | 0.008 | 0.334 | -0.028 | 0.962 | -0.028 | 0.962 |
|  | SDS | 0.023 | 0.124 | 0.015 | 0.183 | -0.010 | 0.730 | -0.010 | 0.707 |
|  | SDH | 0.001 | 0.487 | -0.013 | 0.792 | -0.015 | 0.849 | -0.015 | 0.852 |
|  | SDSSD | 0.029 | 0.040 | 0.025 | 0.068 | -0.002 | 0.556 | -0.002 | 0.542 |
|  | SBS | 0.023 | 0.146 | 0.003 | 0.409 | -0.008 | 0.648 | -0.008 | 0.648 |

Supplementary Table 4 Baseline characteristics of the case population with and without XAJPF

|  | Non-XAJPF group (n=132) | XAJPF group (n=273) | P value |
| --- | --- | --- | --- |
| Gender, n(%) |  |  | 0.127 |
| Male | 13 (9.85) | 42 (15.38) |  |
| Female | 119 (90.15) | 231 (84.62) |  |
| Age, year | 57.50 (17.00) | 59.00 (16.50) | 0.194 |
| Age, n(%) |  |  | 0.534 |
| <60 | 73 (55.30) | 142 (52.01) |  |
| ≥60 | 59 (44.70) | 131 (47.99) |  |
| BMI, kg/m^2^ | 22.07 (4.77) | 22.31 (4.24) | 0.870 |
| Smoking, n(%) | 10 (7.58) | 35 (12.82) | 0.115 |
| Drinking, n(%) | 25 (18.94) | 49 (17.95) | 0.809 |
| Course of disease, year | 8.00 (15.50) | 9.00 (16.00) | 0.851 |
| CCI | 4.00 (2.00) | 4.00 (2.00) | 0.288 |

Supplementary Table 5 Baseline characteristics of low-exposure and high-exposure populations according to XAJPF use categories

|  | Low-exposure group (n=201) | High-exposure group (n=72) | P value |
| --- | --- | --- | --- |
| Gender, n(%) |  |  | 0.061 |
| Male | 26 (12.94) | 16 (22.22) |  |
| Female | 175 (87.06) | 56 (77.78) |  |
| Age, year | 59.00 (16.50) | 58.00 (16.50) | 0.895 |
| Age, n(%) |  |  | 0.901 |
| <60 | 105 (52.24) | 37 (51.39) |  |
| ≥60 | 96 (47.76) | 35 (48.61) |  |
| BMI, kg/m^2^ | 22.21 (4.43) | 22.75 (3.60) | 0.649 |
| Smoking, n(%) | 25 (12.44) | 10 (13.89) | 0.752 |
| Drinking, n(%) | 39 (19.40) | 10 (13.89) | 0.295 |
| Course of disease, year | 9.00 (15.50) | 8.00 (17.50) | 0.202 |
| CCI | 4.00 (2.00) | 4.00 (2.00) | 0.890 |

Supplementary Table 6 Sensitivity analysis

| Outcome measures | Variables | Event N / N | Crude model | | Partially adjusted model | | Fully adjusted model | |
| --- | --- | --- | --- | --- | --- | --- | --- | --- |
|  |  |  | OR (95%CI) | P value | OR (95%CI) | P value | OR (95%CI) | P value |
| PF | Non-exposure | 133/227 | Ref. |  | Ref. |  | Ref. |  |
|  | XAJPF exposure | 208/583 | 0.39 (0.29, 0.54) | <0.001 | 0.38 (0.27, 0.52) | <0.001 | 0.37 (0.27, 0.51) | <0.001 |
|  | XAJPF exposure level, P for trend |  |  | <0.001 |  | <0.001 |  | <0.001 |
|  | Low-exposure | 151/436 | 0.37(0.27, 0.52) | <0.001 | 0.36 (0.26, 0.51) | <0.001 | 0.36 (0.26, 0.50) | <0.001 |
|  | High-exposure | 57/147 | 0.45 (0.29, 0.68) | <0.001 | 0.42 (0.27, 0.65) | <0.001 | 0.41 (0.27, 0.64) | <0.001 |
| GH | Non-exposure | 111/227 | Ref. |  | Ref. |  | Ref. |  |
|  | XAJPF exposure | 216/583 | 0.62 (0.45, 0.84) | 0.002 | 0.62 (0.45, 0.85) | 0.003 | 0.60 (0.44, 0.83) | 0.002 |
|  | XAJPF exposure level, P for trend |  |  | 0.008 |  | 0.009 |  | 0.006 |
|  | Low-exposure | 159/436 | 0.60 (0.43, 0.83) | 0.002 | 0.60 (0.43, 0.84) | 0.002 | 0.59 (0.42, 0.82) | 0.002 |
|  | High-exposure | 57/147 | 0.66 (0.43, 1.01) | 0.055 | 0.67 (0.44, 1.02) | 0.064 | 0.65 (0.42, 1.00) | 0.052 |
| VT | Non-exposure | 134/227 | Ref. |  | Ref. |  | Ref. |  |
|  | XAJPF exposure | 267/583 | 0.59 (0.43,0.80) | 0.001 | 0.57 (0.42, 0.79) | 0.001 | 0.56 (0.41, 0.77) | <0.001 |
|  | XAJPF exposure level, P for trend |  |  | 0.003 |  | 0.002 |  | 0.001 |
|  | Low-exposure | 201/436 | 0.59 (0.43, 0.82) | 0.002 | 0.59 (0.43, 0.82) | 0.002 | 0.58 (0.42, 0.81) | 0.001 |
|  | High-exposure | 66/147 | 0.57 (0.37, 0.86) | 0.008 | 0.53 (0.34, 0.81) | 0.003 | 0.51 (0.33, 0.78) | 0.002 |
| MH | Non-exposure | 124/227 | Ref. |  | Ref. |  | Ref. |  |
|  | XAJPF exposure | 246/583 | 0.61 (0.45, 0.83) | 0.001 | 0.57 (0.42, 0.79) | 0.001 | 0.57 (0.41, 0.78) | <0.001 |
|  | XAJPF exposure level, P for trend |  |  | 0.005 |  | 0.002 |  | 0.002 |
|  | Low-exposure | 180/436 | 0.58 (0.42, 0.81) | 0.001 | 0.56 (0.40, 0.78) | 0.001 | 0.55 (0.39, 0.77) | <0.001 |
|  | High-exposure | 66/147 | 0.68 (0.45, 1.03) | 0.067 | 0.61 (0.40, 0.93) | 0.023 | 0.61 (0.40, 0.95) | 0.027 |
| CPRI-RA | Non-exposure | 138/227 | Ref. |  | Ref. |  | Ref. |  |
|  | XAJPF exposure | 267/583 | 0.55 (0.40, 0.75) | <0.001 | 0.54 (0.39, 0.74) | <0.001 | 0.53 (0.38, 0.73) | <0.001 |
|  | XAJPF exposure level, P for trend |  |  | 0.001 |  | 0.001 |  | 0.001 |
|  | Low-exposure | 199/436 | 0.54 (0.39, 0.75) | <0.001 | 0.54 (0.39, 0.75) | <0.001 | 0.53 (0.38, 0.74) | <0.001 |
|  | High-exposure | 68/147 | 0.56 (0.37, 0.85) | 0.006 | 0.52 (0.34, 0.80) | 0.003 | 0.52 (0.33, 0.80) | 0.003 |
| SDS | Non-exposure | 129/227 | Ref. |  | Ref. |  | Ref. |  |
|  | XAJPF exposure | 228/583 | 0.49 (0.36, 0.67) | <0.001 | 0.49 (0.36, 0.67) | <0.001 | 0.47 (0.34, 0.65) | <0.001 |
|  | XAJPF exposure level, P for trend |  |  | <0.001 |  | <0.001 |  | <0.001 |
|  | Low-exposure | 170/436 | 0.49 (0.35, 0.67) | <0.001 | 0.49 (0.35, 0.68) | <0.001 | 0.47 (0.34, 0.66) | <0.001 |
|  | High-exposure | 58/147 | 0.50 (0.33, 0.76) | 0.001 | 0.48 (0.31, 0.74) | 0.001 | 0.47 (0.30, 0.72) | 0.001 |
| SDH | Non-exposure | 134/227 | Ref. |  | Ref. |  | Ref. |  |
|  | XAJPF exposure | 221/583 | 0.42 (0.31,0.58) | <0.001 | 0.43 (0.32, 0.60) | <0.001 | 0.40 (0.29, 0.55) | <0.001 |
|  | XAJPF exposure level, P for trend |  |  | <0.001 |  | <0.001 |  | <0.001 |
|  | Low-exposure | 159/436 | 0.40 (0.29, 0.55) | <0.001 | 0.41 (0.29, 0.57) | <0.001 | 0.37 (0.27, 0.53) | <0.001 |
|  | High-exposure | 62/147 | 0.51 (0.33, 0.77) | 0.002 | 0.51 (0.33, 0.78) | 0.002 | 0.48 (0.31, 0.74) | 0.001 |
| SDSSD | Non-exposure | 117/227 | Ref. |  | Ref. |  | Ref. |  |
|  | XAJPF exposure | 241/583 | 0.66 (0.49, 0.90) | 0.009 | 0.67 (0.49, 0.91) | 0.011 | 0.62 (0.45, 0.86) | 0.004 |
|  | XAJPF exposure level, P for trend |  |  | 0.032 |  | 0.039 |  | 0.016 |
|  | Low-exposure | 179/436 | 0.66 (0.47, 0.90) | 0.010 | 0.67 (0.48, 0.93) | 0.017 | 0.62 (0.44, 0.87) | 0.006 |
|  | High-exposure | 62/147 | 0.69 (0.45, 1.04) | 0.077 | 0.65 (0.43, 1.00) | 0.050 | 0.63 (0.40, 0.97) | 0.037 |

Notes: Crude model: unadjusted; Partially adjusted model: adjusted for gender, age, BMI, smoking, drinking, course of disease, and CCI; Fully adjusted model: adjusted for the terms in the partially adjusted model and for all baseline inflammatory indicators including NLR, SIRI, ESR, hs-CRP, RF, and CCP.
